# Combined rTMS/fMRI Studies: An Overlooked Resource in Animal Models

**DOI:** 10.3389/fnins.2018.00180

**Published:** 2018-03-23

**Authors:** Bhedita J. Seewoo, Sarah J. Etherington, Kirk W. Feindel, Jennifer Rodger

**Affiliations:** ^1^Experimental and Regenerative Neurosciences, School of Biological Sciences, The University of Western Australia, Perth, WA, Australia; ^2^Centre for Microscopy, Characterization and Analysis, Research Infrastructure Centers, The University of Western Australia, Perth, WA, Australia; ^3^School of Veterinary and Life Sciences, Murdoch University, Perth, WA, Australia; ^4^School of Biomedical Sciences, University of Western Australia, Perth, WA, Australia; ^5^Brain Plasticity Group, Perron Institute for Neurological and Translational Research, Perth, WA, Australia

**Keywords:** translational studies, non-invasive animal models, neuromodulation, rTMS, fMRI, resting-state, functional connectivity

## Abstract

Repetitive transcranial magnetic stimulation (rTMS) is a non-invasive neuromodulation technique, which has brain network-level effects in healthy individuals and is also used to treat many neurological and psychiatric conditions in which brain connectivity is believed to be abnormal. Despite the fact that rTMS is being used in a clinical setting and animal studies are increasingly identifying potential cellular and molecular mechanisms, little is known about how these mechanisms relate to clinical changes. This knowledge gap is amplified by non-overlapping approaches used in preclinical and clinical rTMS studies: preclinical studies are mostly invasive, using cellular and molecular approaches, while clinical studies are non-invasive, including functional magnetic resonance imaging (fMRI), TMS electroencephalography (EEG), positron emission tomography (PET), and behavioral measures. A non-invasive method is therefore needed in rodents to link our understanding of cellular and molecular changes to functional connectivity changes that are clinically relevant. fMRI is the technique of choice for examining both short and long term functional connectivity changes in large-scale networks and is becoming increasingly popular in animal research because of its high translatability, but, to date, there have been no reports of animal rTMS studies using this technique. This review summarizes the main studies combining different rTMS protocols with fMRI in humans, in both healthy and patient populations, providing a foundation for the design of equivalent studies in animals. We discuss the challenges of combining these two methods in animals and highlight considerations important for acquiring clinically-relevant information from combined rTMS/fMRI studies in animals. We believe that combining rTMS and fMRI in animal models will generate new knowledge in the following ways: functional connectivity changes can be explored in greater detail through complementary invasive procedures, clarifying mechanism and improving the therapeutic application of rTMS, as well as improving interpretation of fMRI data. And, in a more general context, a robust comparative approach will refine the use of animal models of specific neuropsychiatric conditions.

## Introduction

An exciting approach for the treatment of neuropsychiatric conditions is to use neuronal activity itself to encourage repair and improve brain function. This method can be used as an adjuvant with other interventions and might prove to be more effective and specific than a pharmacological approach, which often has side-effects and might not induce lasting changes. Several lines of evidence suggest that dysfunctional connectivity within specific neural networks may underpin many neurological and psychiatric conditions (Seeley et al., 2009; van den Heuvel and Hulshoff Pol, 2010). Transcranial magnetic stimulation (TMS) is a non-invasive neuromodulation technique that uses magnetic fields to induce electrical currents in the brain, thereby modulating neuronal activity, and networks (Barker and Freeston, 2007; Wassermann and Zimmermann, 2012). TMS works according to the principle of electromagnetic induction: pulses of current flowing through a TMS coil generate a controllable, pulsatile magnetic field that passes into the brain unimpeded by skin, muscle or skull (basic principles described in Walsh, 1998). This time-dependent magnetic field induces transient electrical currents within the brain. TMS is able to stimulate the human brain and deep peripheral nerves without causing pain because current is not induced in the skin, i.e., pain fiber nerve endings are not activated. This lack of discomfort enables the technique to be used readily on patients and volunteers for research and therapeutic purposes (Barker and Freeston, 2007).

Repetitive transcranial magnetic stimulation (rTMS) delivers trains of closely spaced pulses to the brain to induce transient modulation of neural excitability and brain function. Although transient, the modulation can outlast the stimulation period leading to long-term changes in synaptic plasticity and behavior (for review, see Lenz and Vlachos, 2016). George et al. (1995) were the first to use rTMS as a treatment for depression and its efficacy in medication-resistant patients has been validated by numerous clinical trials (Gaynes et al., 2014). In 2008, an rTMS device developed by Neuronetics was approved by the Food and Drug Administration in the United States for the treatment of patients with major depressive disorder who are resistant to at least one antidepressant drug (O'Reardon et al., 2007). rTMS has since been shown to have therapeutic potential for a range of psychiatric disorders, including unipolar (Xia et al., 2008; Gaynes et al., 2014) and bipolar depression (Xia et al., 2008), schizophrenia (Dlabac-de Lange et al., 2010), obsessive-compulsive disorder (Jaafari et al., 2012), and post-traumatic stress disorder (Clark et al., 2015) as well as in neurological conditions such as Parkinson's disease (Arias-Carrión, 2008), dystonia (Machado et al., 2011), tinnitus (Soleimani et al., 2015), epilepsy (Pereira et al., 2016), and stroke (Corti et al., 2012). rTMS has also shown promising results in the treatment of pain syndromes such as migraine (Lipton and Pearlman, 2010) and chronic pain (Galhardoni et al., 2015). However, there is significant inter- and intra-individual variability in the after-effects induced by rTMS and increasing evidence suggests that subject-related variables such as gender, age, exercise, diet, use of neuropharmacological drugs, the state of the subject, and genetic background might affect the stimulation-induced effects of rTMS in both healthy individuals and patient populations (for review, see Ridding and Ziemann, 2010). To improve the safety and efficacy of rTMS in a clinical setting, a better understanding of how rTMS affects the brain is required (Müller-Dahlhaus and Vlachos, 2013).

Animal models have been useful in elucidating some of the mechanisms of rTMS as they allow us to perform invasive studies of molecular and genetic changes that are not ethically possible in humans. These studies have been reviewed extensively elsewhere (e.g., Tang et al., 2015; Lenz and Vlachos, 2016). However, to align the different experimental approaches used in preclinical animal studies (invasive: cellular and molecular outcomes) and in human studies (non-invasive: e.g., TMS and motor-evoked potentials, MEPs; electroencephalography, EEG; optical imaging, positron emission tomography, PET; functional magnetic resonance imaging, fMRI, behavior) is difficult. Although MEPs (electrical signals induced in muscles following cortical stimulation), which are currently the most common outcome measure used in humans, can also be measured in animals (Rotenberg et al., 2010; Sykes et al., 2016), this approach lacks sensitivity and can be applied only to motor cortical areas. The vast majority of TMS research and clinical treatments target non-motor regions such as the prefrontal or sensory cortex (e.g., Schneider et al., 2010; Liston et al., 2014; Jansen et al., 2015; Valchev et al., 2015), with effects that extend to deeper regions that are not accessible to MEPs (e.g., Komssi et al., 2004). Similarly, EEG (e.g., Komssi et al., 2004; Benali et al., 2011) and optical imaging (e.g., Allen et al., 2007; Kozel et al., 2009) have restricted depth of recording and can detect rTMS-induced functional changes only in the most superficial regions of the brain.

The ability to measure whole-brain functional connectivity before and after rTMS is important because rTMS can induce widespread changes both in cortical and subcortical networks. Currently, PET and fMRI are the only techniques capable of measuring functional effects of rTMS in the whole brain. Combined rTMS/PET have been used in humans (e.g., Paus et al., 1997; Kimbrell et al., 1999; Speer et al., 2000; Mintun et al., 2001; Conchou et al., 2009) and animal models (e.g., Gao et al., 2010; Salinas et al., 2013). However, a major disadvantage of PET with regards to safety is the use of radiotracers, exposing subjects to ionizing radiation. A single PET scan using a standard radiotracer dose leads to radiation exposure up to an order of magnitude greater than that received annually from background radiation. Therefore, longitudinal rTMS/PET studies requiring repeated measurements are not ethically feasible. fMRI, on the other hand, does not require the use of ionizing radiation and therefore, is a safe imaging tool appropriate for repeated long-term experiments.

Therefore, in this review, we suggest that fMRI will be a powerful tool amenable to visualizing and comparing rTMS-induced short and long term neural connectivity changes throughout the brain at high spatio-temporal resolutions in both humans and animals. This method could potentially help unravel the physiological processes underlying the rTMS-induced changes in the cortex and in functionally connected brain regions. Comparison of rTMS effects in human and animal studies will also provide insight into the usefulness of animal models in understanding and improving rTMS based treatments for humans. Here, we review findings from combined rTMS/fMRI studies in humans and consider potential insights from, and limitations of, using fMRI in animal rTMS studies.

## Information obtained from combined rTMS and fMRI techniques in humans

### Ability of fMRI to detect network-level effects of rTMS on healthy volunteers

**Figure 1:**
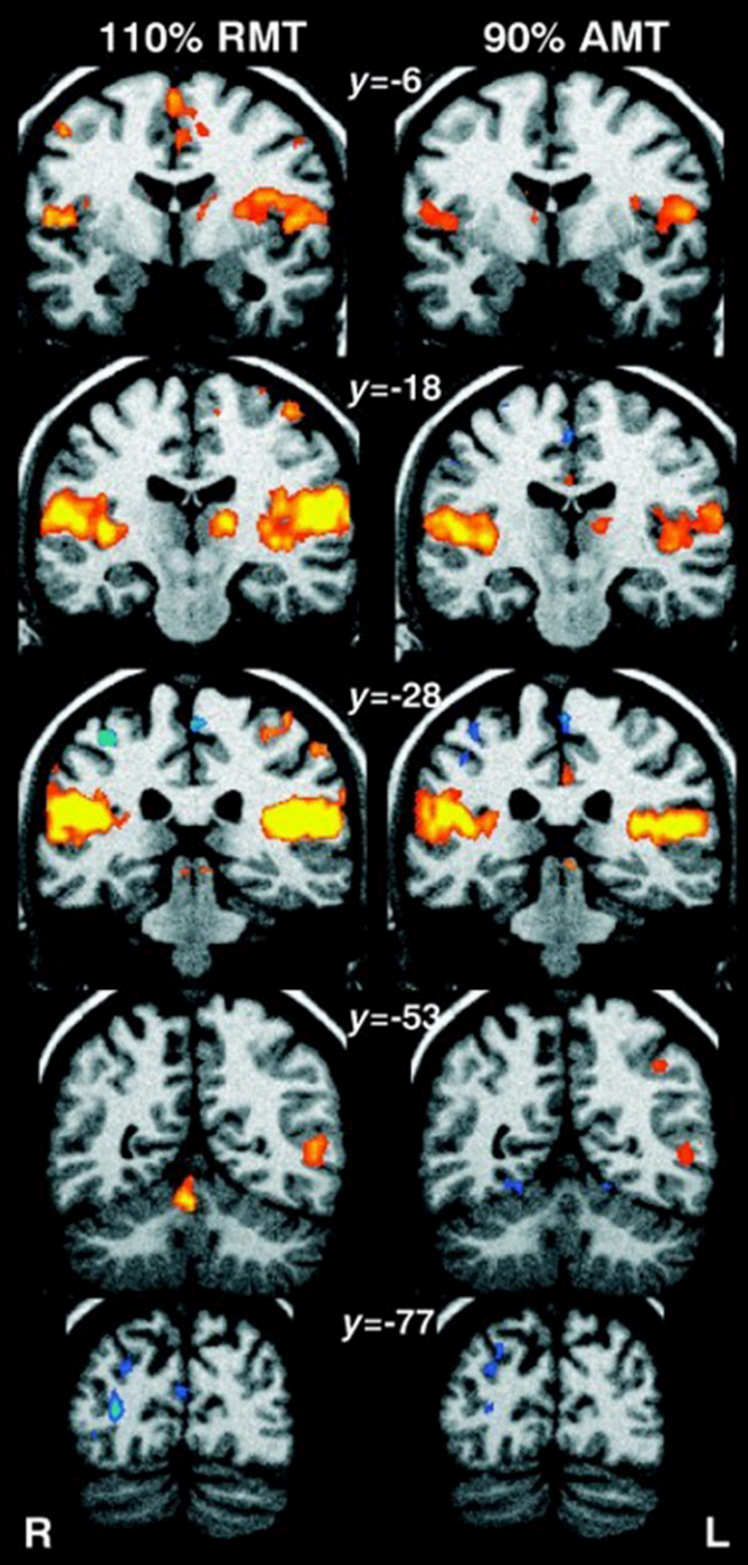
Human fMRI data showing whole-brain effects of **(Left)** high-intensity rTMS at 110% resting motor threshold (RMT), and **(Right)** low-intensity rTMS at 90% active motor threshold (AMT). Coronal standard MNI brain sections (Talairach coordinates indicated) with superimposed fMRI results are shown. Areas of significant (*n* = 11, corrected *p* < 0.01) activations during rTMS compared to rest have been colored red-yellow, and decrease in fMRI signal in blue. Suprathreshold rTMS induced an increase in activity in the stimulated left sensorimotor cortex, medial supplementary and cingulate motor area, auditory cortex, lateral postcentral region, and left thalamus. Decrease in activity was observed in the right sensorimotor cortex and occipital cortex. Subthreshold rTMS produced similar but smaller activations, with no significant changes in the stimulated brain region. Image from Bestmann et al. (2004). Permission to reuse Figure 7 from the article was granted by John Wiley and Sons and Copyright Clearance Center through their RightsLink® service on 17th of August 2017.

Bohning et al. (1998) were the first to demonstrate the feasibility of combining TMS and fMRI protocols, by performing TMS stimulation inside an MRI scanner. TMS applied to the primary motor cortex (M1) in humans resulted in a significant increase in activity in M1 as detected by the fMRI scan. Soon after, the same authors demonstrated that there was a significant increase in activity not only in M1 but also in areas distal to the stimulation site (e.g., the contralateral M1 and ipsilateral cerebellum), illustrating the potential of this technique for mapping connectivity patterns between brain areas (Bohning et al., 1999). The ability of rTMS to target both local and remote brain regions was confirmed by Bestmann et al. (2004), who used interleaved rTMS/fMRI to compare different intensities of rTMS (Figure 1). They showed that fMRI can detect effects of rTMS delivered at an intensity that does not elicit a motor response (i.e, MEP). Therefore, fMRI provides a significant improvement in terms of sensitivity and resolution over MEPs.

Since then, a range of combined rTMS/fMRI human studies have been conducted to record the rTMS-induced changes in hemodynamic activity both in healthy subjects and subjects with neurological disorders (Schneider et al., 2010; Fox et al., 2012b). In healthy subjects, for example, fMRI was used to detect plastic changes induced in the brain after 5 Hz rTMS was applied to the right dorsolateral prefrontal cortex (DLPFC) (Esslinger et al., 2014). No change in activation was detected at the stimulation site, but there was increased connectivity within the right DLPFC as well as from the stimulated DLPFC to the ipsilateral superior parietal lobule, which is functionally associated with the right DLPFC during working memory (Esslinger et al., 2014). This increased connectivity was associated with a decrease in reaction time during a working memory task (*n*-back task). These results suggested the presence of rTMS-induced plasticity in prefrontally connected networks downstream of the stimulation site (Esslinger et al., 2014). Similar results were found when Valchev et al. (2015) delivered a continuous train of theta burst stimulation (cTBS) to the left primary somatosensory cortex of healthy volunteers. Functional connectivity between the stimulated brain region and several functionally-connected brain regions, including the dorsal premotor cortex, cerebellum, basal ganglia, and anterior cingulate cortex, decreased. Another study applying high-frequency (10 Hz) rTMS to the right DLPFC in healthy volunteers while passively viewing emotional faces found significant right amygdala activity attenuation when evaluating negatively valenced visual stimuli (Baeken et al., 2010). Taken together, these studies show that rTMS can have widespread effects, not limited to the stimulated brain area and demonstrate that brain stimulation studies and treatment plans need to take network-level effects into account. Although the hippocampus as a deep brain structure is unlikely to be directly modulated by rTMS, which affects only superficial regions immediately beneath the coil, an ipsilateral change in the hippocampus was detected following multiple-session high-frequency (20 Hz) stimulation to the left lateral parietal cortex of healthy adults (Wang et al., 2014). Increased functional connectivity was observed and this change was correlated with improved associative memory performance. The effects of rTMS on these brain regions (e.g., cerebellum, basal ganglia, cingulate cortex, amygdala, hippocampus) are interesting because they have therapeutic implications which will be summarized in section Potential Applications of Combined rTMS/fMRI Studies in Human Diseases, along with more detailed effects of rTMS on functional connectivity in the intact normal state vs. diseased states.

### Activation patterns in healthy vs. diseased states in humans

While fMRI detects changes in brain activity during an active task, resting-state fMRI (rs-fMRI) provides information about connectivity between brain regions at rest, i.e., when no specific stimulus or task is presented. Rs-fMRI detects brain regions whose patterns of spontaneous blood oxygen level dependent (BOLD) contrast fluctuations are temporally correlated when the subject is at rest. These brain regions with coherent spontaneous fluctuations in activity form an organized network called the resting-state network (Biswal et al., 1995). The default mode network (DMN), a resting-state network with a synchronized activity pattern, shows highest activation when the subject is at rest and is deactivated in goal-oriented tasks (Raichle et al., 2001). The DMN has been associated with cognitive performance and is thought to play an important role in neuroplasticity through the consolidation and maintenance of brain functions (Marcotte et al., 2013). For example, a higher resting-state activity within the DMN is hypothesized to favor network efficiency (Kelly et al., 2008), while decreased connectivity between the frontal and posterior DMN brain regions is associated with functional deficits (Davis et al., 2009). Consistent with these hypotheses, patients with neurological and psychiatric disorders show DMN dysregulation compared with healthy individuals (for review, see van den Heuvel and Hulshoff Pol, 2010). Disruptions in functional connectivity between brain regions forming part of the DMN have been implicated, *inter alia*, in patients with conditions like Alzheimer's disease (Greicius et al., 2004), multiple sclerosis (Lowe et al., 2002; Sbardella et al., 2015), autism (Cherkassky et al., 2006; Kennedy et al., 2006), epilepsy (Waites et al., 2006), depression (Greicius et al., 2007), schizophrenia (Bluhm et al., 2007; Whitfield-Gabrieli et al., 2009), aphasia (Marcotte et al., 2013), and addiction (Sutherland et al., 2012; Lerman et al., 2014).

**Figure 2:**
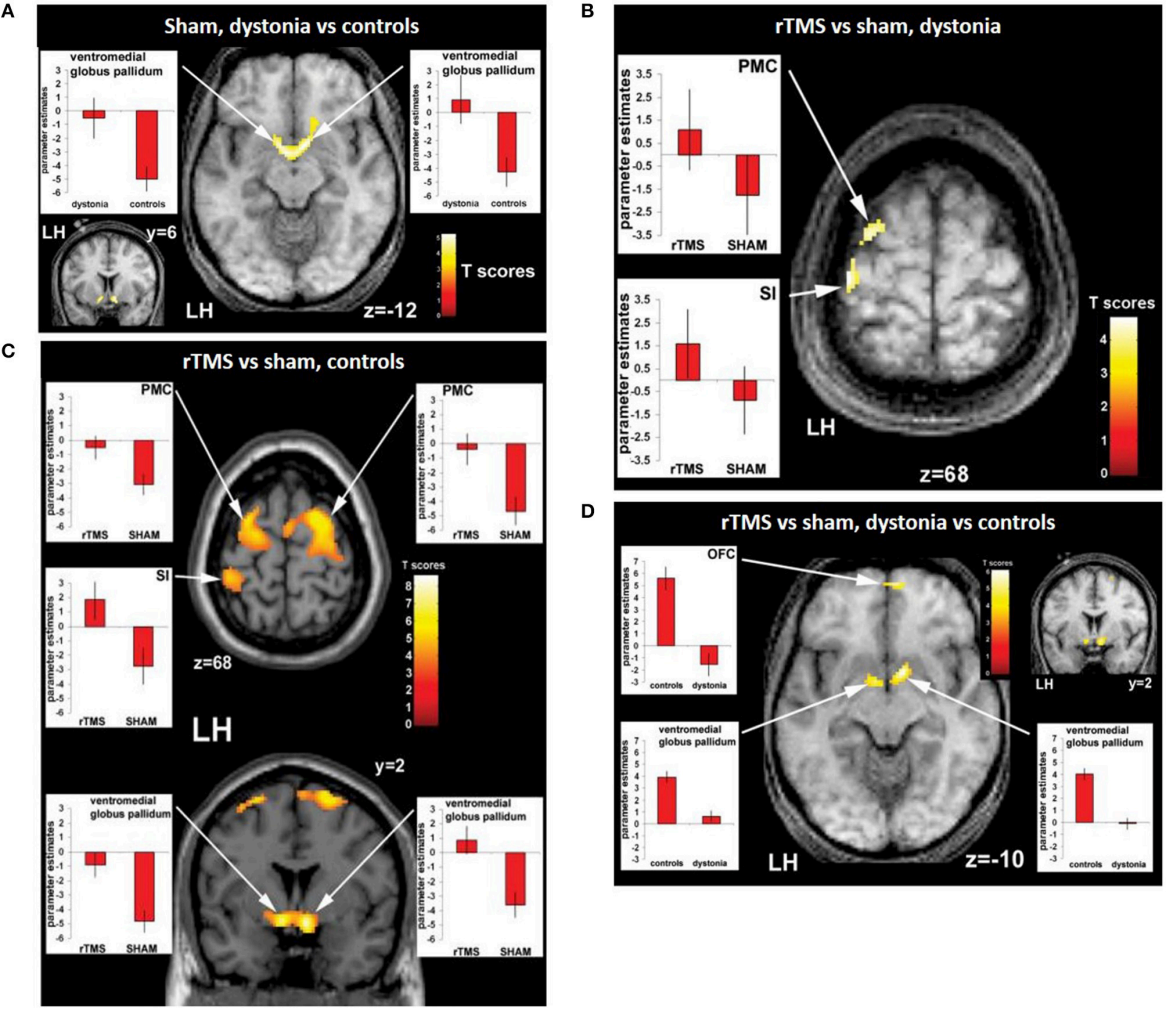
Evidence of functional connectivity abnormalities in patients with dystonia and changes following rTMS. **(A)** Connectivity maps of patients vs. controls during the sham condition showing bilaterally greater activity in the ventromedial pallidum of patients compared to healthy controls. Effects of rTMS (real versus sham condition) on neuronal activity during sensory discrimination task in dystonia patients **(B)** and controls **(C)**. Both patients with dystonia **(B)** and healthy controls **(C)** show relatively greater activity ipsilaterally in the left premotor cortex (PMC) and the left sensorimotor cortex (S1) following after real-rTMS compared to sham stimulation. In addition, there is activation of the ventromedial pallidum bilaterally in healthy controls, but not in patients with dystonia. **(D)** Interaction (group × condition) during rTMS in patients with dystonia compared to controls. Compared to controls, dystonia patients show reduced activity in the left oribtofrontal cortext (OFC) and the ventromedial pallidum bilaterally after real-rTMS compared to sham stimulation **(D)**. Image from Schneider et al. (2010). Permission to reuse Figures 3 and 4 from the article was granted by John Wiley and Sons and Copyright Clearance Center through their RightsLink® service on 13th of February 2018.

Given that the pathophysiology of many psychiatric and neurological disorders is believed to be related to altered neural connectivity and network dynamics, interleaved rTMS/fMRI protocols provide an opportunity to investigate altered patterns of neural activity in these disorders (for review, see Hampson and Hoffman, 2010). The activation patterns in healthy individuals and patients with neurological or psychiatric conditions can be compared after an rTMS session to determine how these patterns are disrupted in the disease state (Hampson and Hoffman, 2010). For example, Schneider et al. (2010) examined the effect of 5 Hz rTMS on the primary somatosensory cortex in patients with dystonia (a condition associated with impaired somatosensory ability) and healthy controls based on their ability to discriminate between two stimulation frequencies applied to the right index finger before and after the rTMS session. An fMRI scan was carried out together with the tactile discrimination task. Without rTMS application, patients showed relative overactivity in the basal ganglia compared to healthy controls (Figure 2A). rTMS led to an improved performance in this task in healthy controls but not in the patients. There was increased activity detected in the stimulated primary somatosensory cortex and bilateral premotor cortex in both groups (Figures 2B,C) but fMRI detected an increase in activity in the basal ganglia in healthy subjects only (Figure 2D), suggesting an abnormal functional connectivity in the cortico-basal network in dystonia. The authors hypothesized that this could be related to altered sensory circuits and sensorimotor integration in patients with dystonia.

Interestingly, rTMS has been shown to modulate functional connectivity in humans, but the direction (increase or decrease in activity) and extent of this modulation depend on the rTMS protocol used, as we review below (Fox et al., 2012b; more recent articles: Popa et al., 2013; Glielmi et al., 2014; Jansen et al., 2015; Li et al., 2016). Using fMRI, increases and decreases in functional activity have been found depending on the stimulated brain region and the frequency of rTMS, allowing insight into how rTMS affects complex brain circuits (Bohning et al., 1999; Kimbrell et al., 1999).

## rTMS protocols and their specific effects in humans

### Simple stimulation protocols

There is considerable evidence from MEP and animal studies that that low-frequency (<5 Hz) rTMS has long-term synaptic depression (LTD) like effects and thereby decreases brain excitability (Klomjai et al., 2015; Wilson and St George, 2016). The inhibitory effect of low-frequency rTMS has been confirmed in studies of the DMN. For example, van der Werf et al. (2010) applied 1 Hz rTMS for two sessions over the left DLPFC of healthy volunteers. The rTMS sessions appeared to decrease resting-state network activity within the DMN, with the reductions happening in the temporal lobes, distant from the stimulated region. More specifically, they found that the hippocampus had reduced activation bilaterally following the application of low-frequency rTMS. They hypothesized that this change in neuronal activity of the hippocampus could arise from a change in cortical excitability or the transcallosal spread of rTMS effects inducing bilateral inhibition. The inhibitory effect of low-frequency rTMS was also confirmed in a resting-state connectivity study between motor regions in healthy individuals (Glielmi et al., 2014). Interestingly, 1 Hz, an inhibitory frequency, is thought to decrease the activity of inhibitory neurones in the stimulated hemisphere, causing a reduction in the inhibitory interhemispheric drive, which in turn leads to an increase in excitability of the contralateral hemisphere. For example, O'Shea et al. (2007) found that even when 1 Hz stimulation over the left dorsal premotor cortex had no effect on behavior, there was a compensatory increase in activity in the right dorsal premotor cortex and connected medial premotor areas. This contralateral effect of 1 Hz rTMS has been utilized to treat patients with stroke by applying low-frequency stimulation to the unaffected hemisphere to decrease transcallosal inhibition of the lesioned hemisphere and consequently improve motor function in such patients. An rTMS/fMRI study by Grefkes et al. (2010) recruited patients with mild to moderate unilateral hand weakness after a first-ever subcortical ischemic stroke in the middle cerebral artery. Each subject underwent a baseline fMRI scan, a post-sham stimulation scan, and a post 1 Hz stimulation scan. rTMS applied over contralesional M1 significantly improved the motor performance of the paretic hand, and the improvement in symptoms was correlated with the functional connectivity results. The fMRI data showed a decrease in negative transcallosal influences from the contralesional M1 and an increase in functional connectivity between the ipsilesional supplementary motor area (SMA) and M1.

In contrast to low-frequency stimulation, high-frequency (≥5 Hz) rTMS has long-term synaptic potentiation (LTP) like effects and increases brain excitability (Klomjai et al., 2015; Wilson and St George, 2016). In patients with stroke, high-frequency rTMS is sometimes applied directly to the affected hemisphere to increase excitability and promote plasticity of the lesioned hemisphere. For example, rs-fMRI demonstrated a bilateral increase in M1 connectivity in such patients after 10 days of 5 Hz rTMS applied ipsilesionally (Li et al., 2016). There was also an increased connectivity between the stimulated ipsilesional M1 and the SMA, bilateral thalamus, contralesional postcentral gyrus, and superior temporal gyrus and decreased connectivity between the stimulated ipsilesional M1 and the ipsilesional postcentral gyrus, M1, middle frontal gyrus, and superior parietal gyrus. An improved interhemispheric functional connectivity was also found in a case study of post-stroke apathy by Mitaki et al. (2016) when 5 Hz rTMS was applied to the SMA of each hemisphere of the patient over the course of two weeks. The improvement in the interhemispheric functional disconnection was correlated with the patient's recovery from post-stroke apathy.

Even though the studies tend to have small sample sizes (Watrous et al., 2013), these results show that understanding the effects of rTMS on multiple brain regions is important and that the effects can be determined to some extent by specific rTMS protocols. Information about the extent to which the functional connectivity within and between different networks can be modulated by different rTMS protocols may prove helpful in the development of treatment options for dysfunctional connectivity.

### Complex stimulation patterns

In contrast to the simple frequencies described above, theta burst stimulation (TBS) uses a composite stimulation pattern, consisting of repeating bursts of stimuli (Larson et al., 1986). Each burst consists of three pulses of stimulation at 50 Hz, and the bursts are repeated at 5 Hz (0.2 s) (for review, see Cárdenas-Morales et al., 2010). This pattern of stimulation is based on the endogenous brain oscillations observed in the hippocampus (Huang et al., 2005), with human hippocampal theta oscillations being at a lower frequency (around 3 Hz) than the hippocampal theta oscillations of rats (8 Hz) (Watrous et al., 2013; Jacobs, 2014). The effect of TBS on the brain depends on the pattern of stimulation (Ljubisavljevic et al., 2015). For example, when cTBS is applied for 40 s (i.e., 600 stimuli) to M1 in humans, there is a decrease in brain excitability (Green et al., 1997). In contrast, when intermittent TBS (iTBS), with a 2 s train of TBS repeated every 10 s for 190 s (i.e., 600 stimuli), there is an increase in brain excitability (Green et al., 1997). Even though recent studies show evidence of substantial inter- and intra-individual variability in response to TBS (Zangen and Hyodo, 2002; Cho et al., 2012), the two main modalities, in general, have opposite effects on brain excitability.

Research on the DMN extends our understanding of the effects of TBS. iTBS applied over the left and right lateral cerebellum in patients with progressive supranuclear palsy for 10 sessions over the course of two weeks lead to an increased signal in the caudate nucleus bilaterally within the DMN (Brusa et al., 2014). iTBS also increased the efficiency of the impaired functional connectivity between the cerebellar hemisphere and the contralateral M1 observed in these patients compared with healthy individuals and patients with Parkinson's disease. The enhanced functional connectivity between the cerebellar hemisphere, the caudate nucleus, and the cortex was accompanied by an improvement of dysarthria in all patients. iTBS was also shown to have a dose-dependent effect on excitability and functional connectivity within the motor system (Nettekoven et al., 2014). When applied over the M1 of healthy volunteers, iTBS increased the resting-state functional connectivity between the stimulated M1 and premotor regions bilaterally. iTBS also increased connectivity between M1 and the ipsilateral dorsal premotor cortex when the number of stimuli was increased. The authors hypothesized that dense connections between M1 and the regions showing increased functional connectivity might facilitate simultaneous stimulation of these interconnected brain areas by the iTBS protocol, thereby modulating the synchrony of the resting activity in those regions.

There have also been studies of the inhibitory action of cTBS on brain activity using fMRI. Following eight sessions of 30 Hz cTBS applied to the SMA over two consecutive days, patients with Tourette syndrome or chronic tic disorder showed a significant reduction in the activity of SMA and left and right M1 activation during a finger-tapping exercise, suggesting inhibition in the motor network. However, improvement in symptoms was not significantly different between test and control subjects, perhaps because of the small sample size. Similar to 1 Hz rTMS, cTBS has been shown to dis-inhibit contralateral targets; in healthy individuals, cTBS application to the right Heschl's gyrus did not induce changes in the stimulated brain region, but significantly increased activity in the contralateral Heschl's gyrus, postcentral gyrus, and left insula and in the bilateral lateral occipital cortex (Andoh and Zatorre, 2012). The mechanisms underlying this interhemispheric interaction are not well understood but could be related to short-term plasticity or compensatory mechanisms to preserve function by increasing the activity of homologous brain regions in the contralateral hemisphere (Andoh and Zatorre, 2012).

In summary, a range of frequencies and stimulation patterns have been tested in human subjects and have specific impacts on brain function and network connectivity. There is significant opportunity to develop other stimulation paradigms systematically, which might have different neuronal effects, and to produce precise and reproducible effects in the brains of patients.

## Potential applications of combined rTMS/fMRI studies in human diseases

### Change in connectivity post rTMS linked to improvement in symptoms

Change in functional connectivity achieved using rTMS as a treatment method can be used to determine the neural mechanisms of improvement in symptoms in patients. For example, a longitudinal study by González-García et al. (2011) used fMRI to investigate the mechanisms by which 25 Hz rTMS (10 trains of 100 pulses) over M1 for three months improves the motor symptoms of patients with Parkinson's disease. rTMS was found to cause an increase in activity in the caudate nucleus during a simple motor task (finger-tapping test). fMRI also showed a decline in activity in the SMA, which was accompanied by an increase in its functional connectivity to the prefrontal areas. These changes substantiated the beneficial effect of rTMS on the symptoms of Parkinson's disease observed in these patients.

Another way to analyze the link between rTMS therapy and improvement in symptoms is to use rs-fMRI to detect the change in functional connectivity after rTMS. Popa et al. (2013) found that the connectivity within both the cerebello-thalamo-cortical network and the DMN was compromised in patients with essential tremor. Application of 1 Hz rTMS for five consecutive days bilaterally over lobule VIII of the cerebellum appeared to have re-established the connectivity in the cerebello-thalamo-cortical network only, and this change in functional connectivity was accompanied by a significant improvement in symptoms. Another study that also used an rTMS/rs-fMRI protocol carried out a whole-brain connectivity analysis to unravel the effect of rTMS on functional connectivity and motor symptoms in patients with multiple system atrophy (Chou et al., 2015). 5 Hz rTMS was applied over the left M1 of such patients for 10 sessions over the course of two weeks. Only the active rTMS group showed a significant improvement in motor symptoms, and these improvements were correlated to the modulation of functional links connecting to the default mode, cerebellar, and limbic networks by high-frequency rTMS. These findings suggest that rTMS can be used to target specific brain networks as a therapy for patients with multiple system atrophy.

### Predicting susceptibility to rTMS therapy

**Figure 3:**
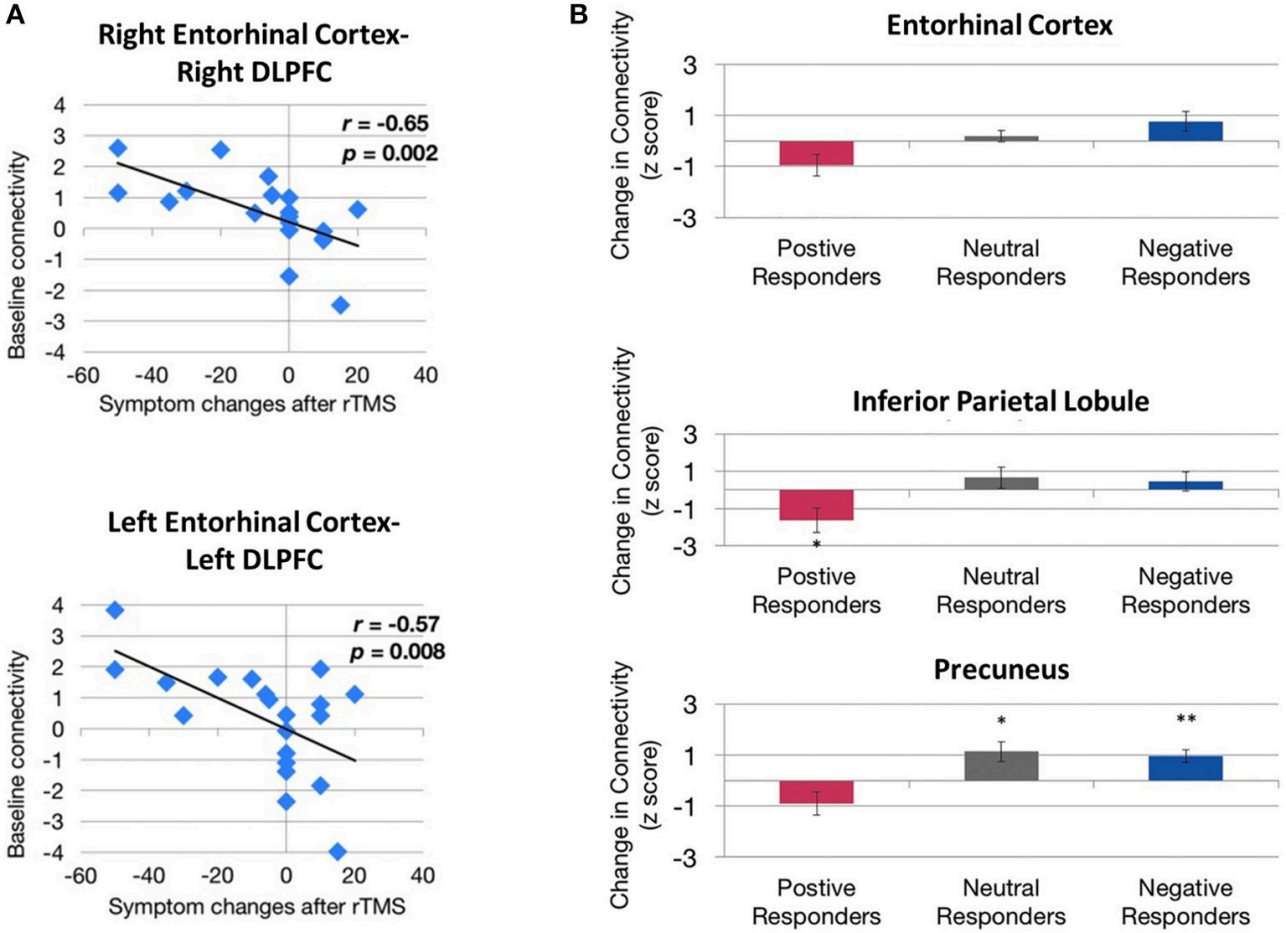
Evidence of correlation between behavioral improvements and functional connectivity before and after rTMS treatment. **(A)** Correlation between baseline entorhinal cortex–dorsolateral prefrontal cortex (DLPFC) functional connectivity and treatment response. Higher baseline functional connectivity exhibited by both right entorhinal cortex–right DLPFC connectivity (top) and left entorhinal cortex–left DLPFC connectivity (bottom) is associated with a greater magnitude of symptom reduction (change < 0) after rTMS. **(B)** Directional effect of functional connectivity changes between the left entorhinal cortex and the right entorhinal cortex (top), right inferior parietal lobule (middle), and the left precuneus (bottom). Functional connectivity between these regions decreased in patients showing improvement in symptoms (positive responders) compared with patients whose symptoms remained unchanged (neutral responders) or worsened (negative responders). ^*^^,^^**^Indicate significant changes for *p* < 0.05 and *p* < 0.01, respectively. Image from Yuan et al. (2017), an open access article distributed under the terms of the Creative Commons Attribution License, which permits unrestricted use, distribution, and reproduction in any medium, provided the original work is properly cited.

More recently, baseline functional connectivity was shown to be a potential predictor of response to rTMS treatment, for example, in Mal de Debarquement Syndrome, a neurological condition representing a persistent false perception of rocking and swaying following exposure to unfamiliar motion patterns (Yuan et al., 2017). Pre- and post-rTMS rs-fMRI were carried out to assess functional connectivity changes as a result of daily rTMS treatment to the DLPFC (1,200 pulses of 1 Hz rTMS over right DLPFC followed by 2000 pulses of 10 Hz rTMS to the left DLPFC) over five consecutive days. A significantly positive baseline functional connectivity between the right DLPFC and the right entorhinal cortex and between the left DLPFC and bilateral entorhinal cortex were identified in patients showing improvement in symptoms following treatment, but not in patients whose symptoms worsened or remained unchanged (Figure 3A). Improvement in symptom severity was correlated with a decrease in functional connectivity between the left entorhinal cortex and posterior DMN regions such as the contralateral entorhinal cortex, the right inferior parietal lobule, and the left precuneus (Figure 3B).

Functional connectivity analysis on pre-treatment fMRI has also been used to predict the response to rTMS treatment in depression. Drysdale et al. (2017) found two groups of functional connectivity features that were linked to specific combinations of clinical symptoms in patients with depression. Anhedonia and psychomotor retardation were primarily linked to frontostriatal and orbitofrontal connectivity features, while anxiety and insomnia were primarily linked to a different group of primarily limbic connectivity features involving the amygdala, ventral hippocampus, ventral striatum, subgenual cingulate, and lateral prefrontal control areas. Testing the abnormalities in these connectivity features, based on their rs-fMRI data, revealed that not all patients with depression had the same functional connectivity patterns. They could be categorized, with high sensitivity and specificity, into four biotypes based on the distinct patterns of dysfunctional connectivity in the frontostriatal and limbic networks, which were most homogeneous within the subtypes and most dissimilar between subtypes. Moreover, patients with these neurophysiological subtypes of depression had different susceptibility to rTMS therapy: after five weeks of high-frequency rTMS delivered to the dorsomedial prefrontal cortex, biotype 1 patients showed a significant response to rTMS therapy (82.5% of that group), and biotype 2 patients were the least responsive (25.0%). This study suggests that heterogeneous symptom profiles in depression could be caused by distinct patterns of dysfunctional connectivity and that categorizing patients into subtypes based on these patterns might enable the prediction of treatment response to rTMS. Two years earlier, Downar et al. (2015) found that the resting-state functional connectivity features of the DLPFC to the subgenual cingulate cortex in patients with major depressive disorder could predict response either to 10 Hz or iTBS rTMS therapy. The role of subgenual cingulate connectivity in patients with depression has been indicated in several other studies (Greicius et al., 2007; Fox et al., 2012a; Liston et al., 2014; Hopman et al., 2017). More recent studies have since confirmed that it is possible to predict the response to iTBS or 10 Hz rTMS using resting-state functional connectivity between ventral striatum and bilateral frontal pole, as well as between the left DLPFC and the left anterior cingulate cortex (Dunlop et al., 2017). Therefore, rs-fMRI potentially could be used to select optimal rTMS parameters for the treatment of patients with depression.

### Other outcomes

Combining fMRI and rTMS has proven useful for investigating a range of other neural functions, providing evidence supporting the following: the functional relevance of the parietal cortex for visuospatial functions (Sack et al., 2002); a link between neural activity in the left inferior prefrontal cortex and episodic memory formation (Köhler et al., 2004); the involvement of premotor cortical areas in speech perception (for review, see Iacoboni, 2008); the presence of functional asymmetry highlighting interhemispheric differences in the auditory network (Andoh et al., 2015); and enabled targeting of stimulation based on the individual's MRI anatomy, functional connectivity results or activated brain regions during specific tasks (e.g., Sack et al., 2002; Fitzgerald et al., 2009; Eldaief et al., 2011; Binney and Lambon Ralph, 2015; Nierat et al., 2015; Valchev et al., 2015).

## Need for animal rTMS/fMRI studies

The induction of plasticity as described in previous sections has been the driving force behind clinical studies of rTMS as a potential treatment of neurological and psychiatric conditions. However, even in neurologically normal subjects, the variability in response to rTMS is high (Maeda et al., 2000). Increasing evidence suggests that rTMS may not induce reliable and reproducible effects, therefore limiting the current therapeutic usefulness of this technology (for review, see Ridding and Ziemann, 2010). Animal models give us the opportunity to mitigate the confounding effects of variability in studies by controlling for gender, age, diet, drugs, genetic background, and the time at which experiments are carried out. The relative importance of these contributing factors can then be identified using animal studies (e.g., state-dependent variability explored in Pasley et al., 2009).

**Figure 4:**
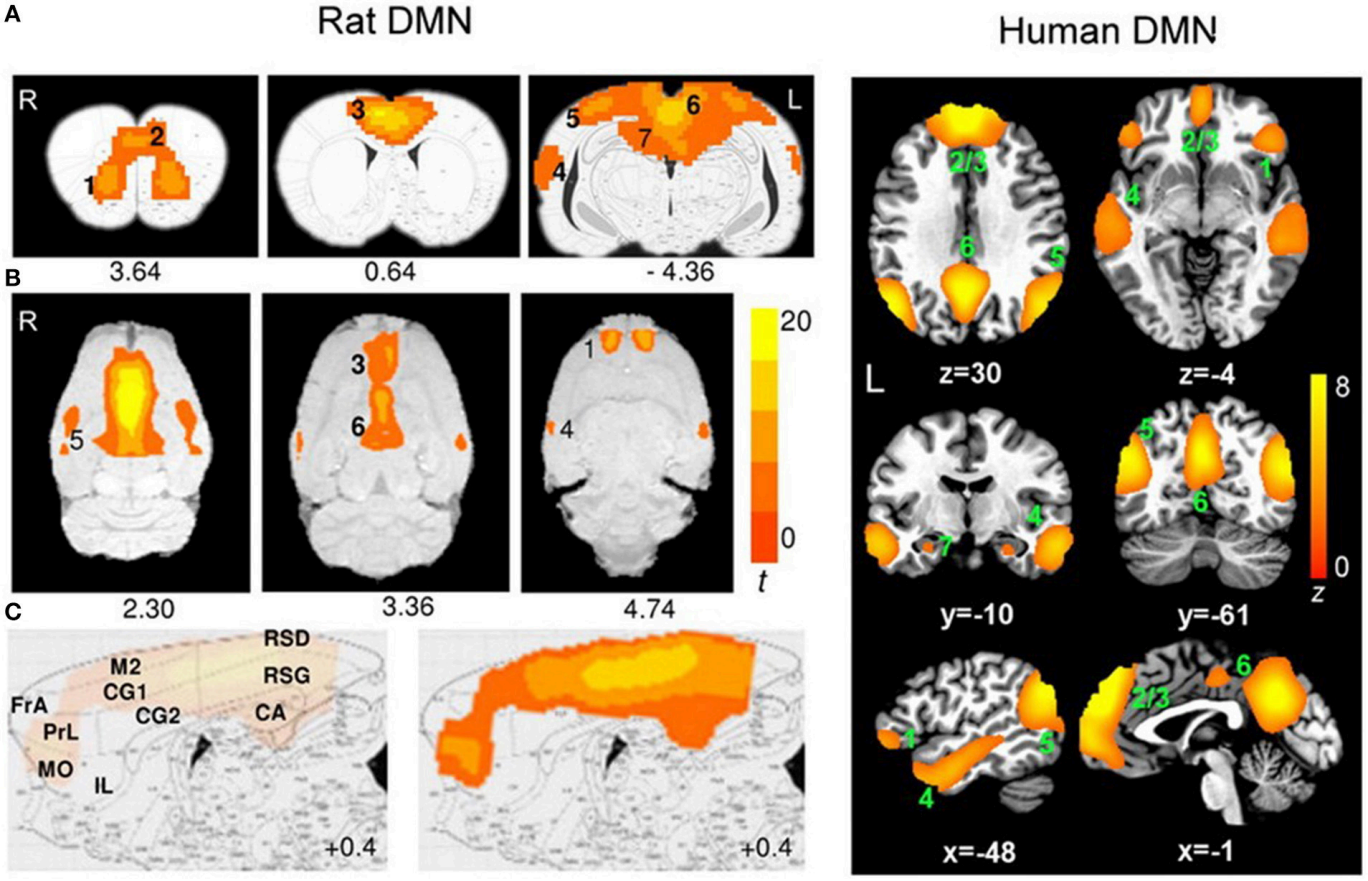
Evidence of translatability of fMRI studies: similar brain regions forming part of the default mode network (DMN) in the rat (Paxino's atlas coordinates indicated) and human (Talairach coordinates indicated) brains. Connectivity maps are shown in the coronal **(A)**, axial **(B)**, and sagittal **(C)** planes. Significant clusters for rat DMN (left) include: 1, orbital cortex; 2, prelimbic cortex (PrL); 3, cingulate cortex (CG1, CG2); 4, auditory/temporal association cortex (Au1, AuD, AuV, TeA); 5, posterior parietal cortex; 6, retrosplenial cortex (corresponding to the posterior cingulate cortex in humans); 7, hippocampus (CA1). The sagittal plane (medial–lateral: +0.4 mm) also shows: FrA, frontal association cortex; MO, medial orbital cortex; RSG/RSD, granular/dysgranular retrosplenial cortex. Color bar indicates t scores (*n* = 16, *t* > 5.6, corrected *p* < 0.05). Significant clusters for human DMN (right) include: 1, orbital frontal cortex; 2/3, medial prefrontal cortex/anterior cingulate cortex; 4, lateral temporal cortex; 5, inferior parietal lobe; 6, posterior cingulate cortex; 7, hippocampus/parahippocampal cortex. Color bar indicates z scores (*n* = 39, *z* > 2.1, corrected *p* < 0.05). Image adapted from Lu et al. (2012), an open access article under the creative commons license and can be republished without the need to apply for permission provided the material is cited correctly and is republished under the same license.

Interleaving rTMS and fMRI has opened doors to many possibilities in the clinical setting. However, there have been no reports of animal studies using those same techniques. fMRI in rodent research is becoming increasingly popular because of its high translatability. Moreover, rodent rs-fMRI studies have confirmed that rodents possess a DMN similar to humans despite the distinct evolutionary paths of rodent and primate brains (Figure 4; Lu et al., 2012). Because rodents are widely used as preclinical models of neuropsychiatric disease (e.g., Tan et al., 2013; Yang et al., 2014, 2015; Zhang et al., 2015; Kistsen et al., 2016), a thorough understanding of how rTMS affects the rodent neural networks is of particular importance for both interpreting rodent fMRI data and translating findings from humans to animals and back again.

### Future experiments: linking functional connectivity changes post rTMS to genetic/molecular changes

Animal models provide a unique opportunity to combine techniques: changes in functional connectivity between spatially separated brain regions caused by long-term modulation of network dynamics by rTMS, can be studied in parallel with changes in behavioral measures, and followed by invasive procedures to detect cellular and molecular changes, all within the same animal. As shown in human rTMS/fMRI studies, the functional connectivity effects of rTMS are distributed across the whole brain (Figure 1). But, even when in-depth studies of the molecular mechanisms are carried out (e.g., quantification of gene expresssion in multiple brain regions using low-density PCR arrays by Ljubisavljevic et al., 2015), how these molecular changes from animal studies underpin functional connectivity changes described in humans remains unclear. Future studies should aim to identify brain regions affected by the stimulation in animals using fMRI and then rTMS mechanisms further elucidated in the same individuals, for example by measuring changes in gene expression in the corresponding brain regions postmortem. Such correlational approaches will provide a compelling view of how rTMS affects the brain at systems levels.

### Future experiments: linking functional connectivity changes post rTMS to structural changes

The induction of brain plasticity has been the driving force behind clinical studies of rTMS as a potential treatment of neurological and psychiatric conditions. A more detailed understanding of how rTMS treatment leads to long-term modulation of network dynamics will be essential for the interpretation of neuropsychological and cognitive effects of rTMS in a clinical context. Combined rTMS/fMRI studies in animals can be used in longitudinal studies to investigate the long-term effects of rTMS. For example, fMRI can be used to measure cumulative effects of repeated rTMS delivery, following which fluorescent dye tracers can be injected into brain to anatomically label neuronal pathways of interest identified by fMRI. Conducting neuronal tract tracing after an fMRI study could elucidate whether long-term treatment with rTMS can eventually elicit changes in fiber tracts in the brain. Analyzing functional connectivity and anatomical connectivity within the same animals will provide information about how observed functional changes correlate with the structural changes detected at a cellular level. Although there is a general trend toward functional and structural connectivity being strongly associated, there are some mismatches within the DMN whereby regions showing high correlation have low fiber connectivity (Hsu et al., 2016). Therefore, future studies to investigate whether the effect of rTMS on functional connectivity is related to fiber connectivity are warranted.

### Future experiments: combined rTMS/fMRI studies using animal models

An important recent development in animal research has been the use of rs-fMRI for the characterization of models of neuropsychiatric disorders. Studies have revealed abnormal functional connectivity patterns in animal models due to pharmacological modulations or genetic manipulations which mimic connection abnormalities observed in corresponding human disorders (Jonckers et al., 2015; Gozzi and Schwarz, 2016; Gorges et al., 2017). These animal models represent a powerful tool to understand the neurobiological basis of the reported ability of rTMS to assist in remediating functional dysconnectivity observed in human disorders. First, fMRI can be used to identify the abnormal functional connectivity in an animal model specific to a neurological disorder. For example, the Wistar-Kyoto rat, an accepted model for depression which has shown resistance to acute antidepressant treatment (Lahmame et al., 1997; López-Rubalcava and Lucki, 2000), also shows functional connectivity anomalies between hippocampus, cortical, and sub-cortical regions (Williams et al., 2014), as has been observed in humans with major depressive disorder. Future studies can investigate the effects of rTMS on such animal models. Functional connectivity changes post-rTMS treatment can then be linked to improvement in symptoms (through behavioral tests such as the forced swim test commonly used in depression studies) as well as to any induced molecular and cellular changes (through postmortem analysis). Therefore, extending the use of combined rTMS/fMRI techniques in various types of neurological and psychiatric conditions using appropriate animal models is a promising avenue for understanding the fundamental properties of the functional re-organization in these conditions in a unique way.

While the use of combined rTMS/fMRI techniques in rodent research has great potential because of its high translatability, there are challenges as we review below.

## Challenges associated with rTMS/fMRI studies in animals

### Choice of animal model and development of rTMS coils

A wide range of animal models have been used with the aim of understanding the underlying mechanisms and optimizing the therapeutic applications of rTMS: rats (Tan et al., 2013; Yang et al., 2015; Zhang et al., 2015), mice (Kistsen et al., 2016), guinea pigs (Mulders et al., 2016), rabbits (Guo et al., 2008), felines (Allen et al., 2007; Valero-Cabré et al., 2007), and in a very limited way, non-human primates (Valero-Cabre et al., 2012; Salinas et al., 2013; Mueller et al., 2014). These animal models have contributed considerably to our current understanding of the non-invasive neuromodulatory effects of rTMS (reviewed in Tang et al., 2015). However, the variation in brain structure and the smaller sized brains of non-human animals present a fundamental challenge in the application of rTMS and interpretation of its effects.

**Figure 5:**
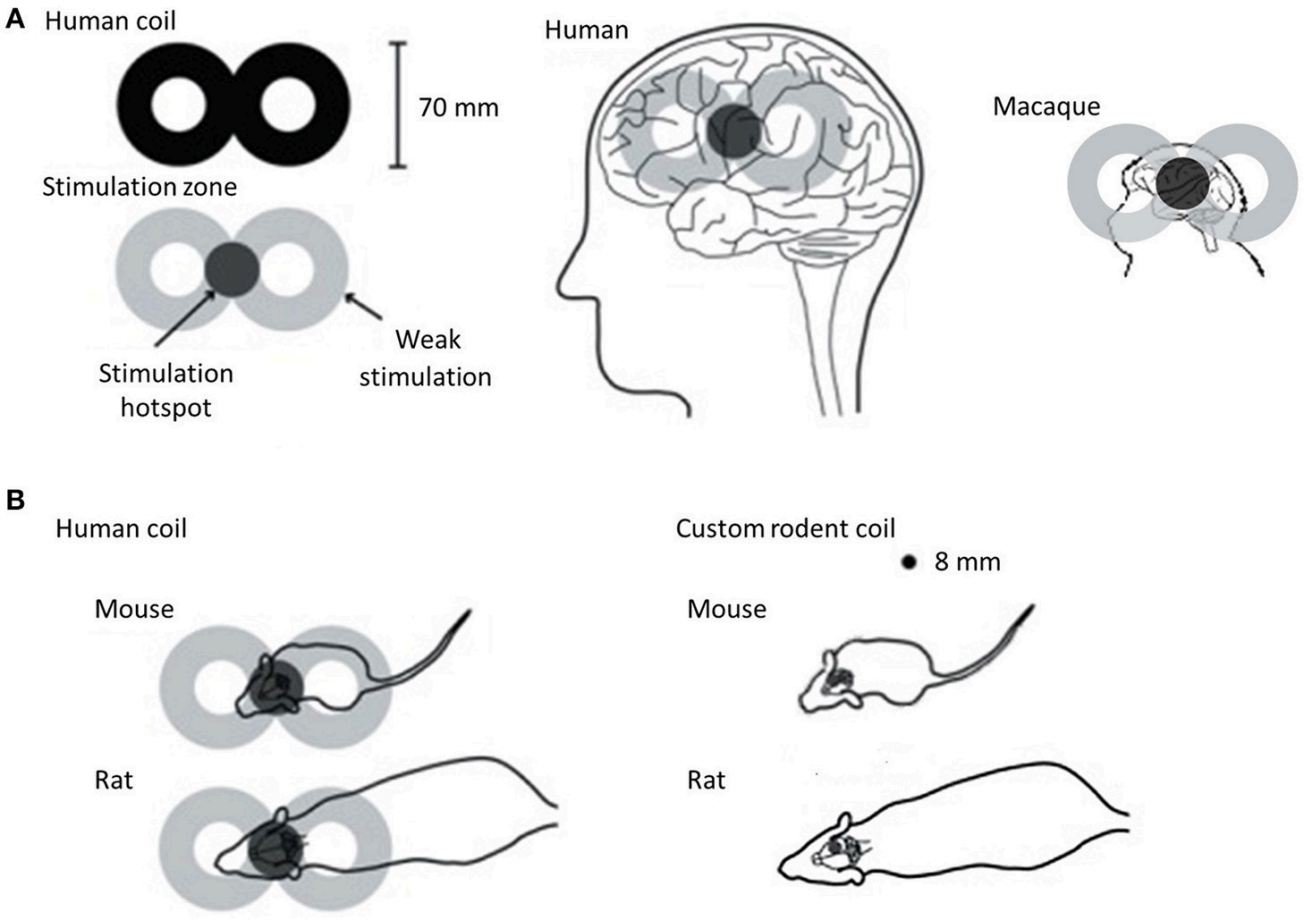
rTMS coil size with respect to brain sizes of humans, macaque and rodents. For all panels, coils are shown in black and the approximate location of the induced current is in gray. **(A)** Typical human “figure of eight” coil with stimulation hotspot at the intersection of the loops in dark gray showing the “focal” stimulation in humans and macaques. When applied to the head, the hotspot is positioned over the target brain region, but the rest of the brain also receives stimulation, albeit at lower intensity. However, when this human coil is applied in rodents **(B)**, the hotspot is no longer focal relative to the target, but rather stimulates the entire head and an appreciable portion of the body (left). This reduces the efficiency of magnetic induction and changes the properties of the induced current. To address this problem, custom-made round coils (right) have been used to deliver focal stimulation in rodents (Rodger et al., 2012; Makowiecki et al., 2014; Tang et al., 2016). Although these deliver low intensity magnetic fields, the induced current is fully contained within the brain, increasing efficiency of induction. The coils are small enough to stimulate one hemisphere in both mice and rats. Image adapted from Rodger and Sherrard (2015), an open-access article distributed under the terms of the Creative Commons Attribution-Noncommercial-Share Alike 3.0 Unported, which permits unrestricted use, distribution, and reproduction in any medium, provided the original work is properly cited.

Non-human primates are clearly the closest to humans in terms of brain structure and size and have the advantage that they can be taught behavioral tasks similar to those used in human rTMS studies. However, the high cost of using non-human primates limit opportunities for invasive studies in large numbers of animals. Rodents, on the other hand, although they are the most commonly used laboratory animals, have important differences in brain structure compared to humans. Their smooth cortex possesses a very different geometry to the highly folded human cortex and this is an important consideration because the characteristics of the electric field induced by rTMS are predicted to be influenced by the orientation of the tissue relative to the coil (Opitz et al., 2011). In addition to differences in brain structure, the small size of the rodent brain presents a serious challenge. In humans, rTMS is most commonly delivered using a coil shaped like a “figure-of-eight” (Figure 5). This configuration provides a stimulation hotspot at the intersection of the loops that can be positioned over the target brain region to provide focal stimulation. Adjacent brain areas also receive stimulation but at much lower intensity. In most animal models, even the smallest commercially available rTMS coil results in a different ratio between head size and coil size from that in humans, reducing stimulation focality and efficiency (Rodger and Sherrard, 2015). For example, using a standard, human-sized “figure-of-eight” coil in mice stimulates the entire brain and often an appreciable portion of the body (Figure 5). The discrepancy in size, therefore, precludes easy interpretation and translation of animal results into clinical applications. Many rodent studies are therefore compromised by the use of large, human-scale coils to deliver rTMS (e.g., Yang et al., 2014, 2015; Zhang et al., 2015), and some groups have used miniaturized rTMS coils in order to more closely mimic focal human rTMS in rodents. For example, custom-made 8 mm diameter round coils have been used to stimulate one hemisphere in both mice and rats (Rodger et al., 2012; Makowiecki et al., 2014), with the induced current fully contained within the brain, increasing the efficiency of induction. However, there is a heat dissipation problem when using small round coils (Cohen and Cuffin, 1991; Wassermann and Zimmermann, 2012), limiting the intensity of the magnetic field to levels roughly 10–100 times lower than those commonly applied in humans (low-intensity; LI-rTMS) (Rodger and Sherrard, 2015).

Despite the existing inability to deliver the same magnetic field parameters in humans and in animals with small brains (Rodger and Sherrard, 2015), beneficial effects are seen in animal models of neurological disease using human rTMS coils (e.g., Tan et al., 2013; Yang et al., 2015; Zhang et al., 2015; Kistsen et al., 2016) and small LI-rTMS coils (Makowiecki et al., 2014; Clarke et al., 2017), suggesting that both approaches have therapeutic potential. Because fMRI can detect both high and low-intensity rTMS effects in the brain, this technique will be useful in establishing a direct comparison of different intensity magnetic field effects in human and animal brains. This information is imperative if we are to define the best clinical protocols for rTMS.

### Use of anesthetics in animal fMRI studies

Although fMRI can be performed on awake, normally behaving animals that have been extensively trained and habituated (Brydges et al., 2013; Kenkel et al., 2016), one advantage of rs-fMRI is that being a task-free technique, functional connectivity can be investigated in anesthetized animals without relying on, or being confounded by, behavior. In human studies, the physiological condition of the subject is assumed to be relatively constant throughout an fMRI scan session (Pan et al., 2015). On the other hand, in animal fMRI, the use of anesthesia is generally required to immobilize the animal and reduce stress (Vincent et al., 2007; Pan et al., 2015). Isoflurane is the anesthetic of choice for repeated long-term experiments because of its ease of use and control, and rapid reversibility (Masamoto and Kanno, 2012). However, anesthetics, including isoflurane, might confound the imaging results as they may cause alterations in neural activity, vascular reactivity, and neurovascular coupling. Isoflurane decreases excitatory and increases inhibitory transmission, causing an overall suppression of neural activity, most likely by modulating the intracellular concentration of calcium (Gomez and Guatimosim, 2003; Ouyang and Hemmings, 2005). As such, the ability of high-frequency rTMS to depolarise neurons is impaired in the presence of isoflurane (Gersner et al., 2011). Additionally, isoflurane, being a GABAergic anesthetic, induces vasodilation through the activation of ATP-sensitive potassium channels of smooth muscle cells in cerebral arteries (Ohata et al., 1999; Pan et al., 2015). Vasodilation leads to an increase in cerebral blood flow, which can be interpreted as an increase in activity. Moreover, the use of an isoflurane-only anesthetic regime has been reported to decrease inter-thalamic connectivity, thalamo–cortical connections, and DMN–thalamic network connections (Bukhari et al., 2017). These potential confounding effects of isoflurane should be taken into account when interpreting findings.

An alternative to general anesthesia is sedation, for example using medetomidine or its active enantiomer, dexmedetomidine. These drugs lack the dose-dependent vasodilation and neural suppression observed with isoflurane use. However, being α2-adrenergic agonists, they activate α2-adrenergic receptors and cause a decrease in cerebral blood flow, potentially because of increased vascular resistance via cerebral vasoconstriction (Prielipp et al., 2002). Moreover, there is a potential issue with prolonged studies because the dose of medetomidine needs to be increased after 2 h to maintain sedation (Pawela et al., 2009). Furthermore, the use of a medetomidine-only anesthetic regime produced decreased inter-cortical connectivity (Bukhari et al., 2017).

Interestingly, using a combination of low-dose isoflurane and medetomidine appears to mitigate some of these factors. This anesthetic combination not only allows stable sedation for over four hours (Lu et al., 2012) but also maintains strong inter-cortical and cortical-subcortical connectivity (Grandjean et al., 2014; Bukhari et al., 2017). The BOLD signal was determined to be maximally stable approximately 90 min after the initiation of medetomidine infusion, suggesting that fMRI data should be collected at this time (Lu et al., 2012). Moreover, data were reproducible from repeated fMRI experiments on the same animal one week apart (Lu et al., 2012). The high reproducibility and minimal impact on network connectivity of the medetomidine/isoflurane combination anesthesia make it a preferred anesthetic regime for prolonged and longitudinal rs-fMRI studies in rodents.

### Rs-fMRI studies in rodents—challenges in data analysis

As with human data, rodent rs-fMRI data need to undergo extensive pre-processing prior to analysis. Rodent studies have used a variety of software packages such as Statistical Parametric Mapping (SPM) (e.g., Jonckers et al., 2011), Analysis of Functional NeuroImages (AFNI) (e.g., Hsu et al., 2016), BrainVoyager (e.g., Hutchison et al., 2010), and Functional MRI of the Brain (FMRIB) Software Library (FSL) (e.g., Tambalo et al., 2015), which were designed for human fMRI data. Therefore, modifications to the data format and pre-processing steps are often necessary to undertake analysis of rodent brain data. For example, the field of view can be altered by up-scaling the voxel sizes by a factor of 10 to be closer to the size of a human brain (Tambalo et al., 2015). Likewise, because of the smaller size of the rodent brain, higher spatial smoothing may be required in animal fMRI data to increase the signal-to-noise ratio without reducing valid activation. Temporal band-pass filtering can also be used to reduce hardware noise, low-frequency signal drifts, and some artifacts caused by cardiac rhythm and respiration, as well as thermal noise (for review, see Pan et al., 2015). Recently, Zerbi et al. (2015) described the use of single-session independent component analysis (ICA) in the FSL for significantly improved artifact reduction in rs-fMRI rodent data. This method removes signals from common sources, for example breathing, which can be isolated into separate components and removed as noise from the data. Additionally, because of the difference in the shape of human and animal brains, skull-stripping—a fully-automated step in human fMRI data pre-processing required to prevent extra-brain matter from interfering with the results—is still largely performed through manual segmentation in animal studies (Sierakowiak et al., 2015; Zerbi et al., 2015). Before analysis, normalization to map functional networks onto a common space is necessary to allow for comparison across subjects or groups. However, because not all rodent strains have an available standard template or atlas for co-registration, some studies acquire and use high-resolution structural images or group-averaged images as a common space (Sierakowiak et al., 2015; Zerbi et al., 2015).

Two of the popular methods for rs-fMRI data are seed-based connectivity analysis (e.g., Hutchison et al., 2010; Sierakowiak et al., 2015; Huang et al., 2016) and ICA (e.g., Hutchison et al., 2010; Jonckers et al., 2011; Lu et al., 2012; Zerbi et al., 2015; Hsu et al., 2016). Seed-based correlation, used in the earliest functional connectivity studies, is a hypothesis-driven approach, which is particularly attractive for area-based hypotheses-driven rs-fMRI studies in rodents. The temporal correlation of all voxels within the brain is analyzed relative to user-defined seed voxel or small region of interest (Joel et al., 2011). However, seed-points can differ in their location between studies, which affects the connectivity patterns considerably and renders comparisons between studies difficult. In addition, this method is not suitable for exploratory analyses. In contrast, the use of data-driven ICA enables identification of networks of functional connectivity within the entire brain without *a priori* knowledge, and thus is a less biased approach (Cole et al., 2010). Moreover, this approach might improve reproducibility because there is no (arbitrary) seed selection (Rosazza et al., 2012). However, the results might depend on the number of components used. Despite the rodent DMN producing similar spatial patterns and being robust irrespective of the number of components (Lu et al., 2012), the number of components chosen can impact on the ease of analysis. For example, Jonckers et al. (2011) and Hsu et al. (2016) chose 15-components in ICA over ICAs repeated with a higher number of components to avoid splitting of the DMN or splitting of some brain regions into different components. Therefore, despite ICA becoming increasingly popular for the analysis of rodent rs-fMRI data, challenges remain as there is no set protocol for selecting the number of components.

## Summary

Considered together, the human studies discussed in this review demonstrate the broad relevance and significance of the results from combined rTMS/fMRI protocols. Results from human studies to date *inter alia* have permitted the characterization of corrupted networks in diseased states, as well as intriguing glimpses into how alterations in functional connectivity patterns after rTMS are correlated with improvements in symptoms. There is also exciting evidence that functional connectivity can be used to predict responses to rTMS treatment with high reliability.

Although methodological challenges remain in the use of brain imaging techniques in rodents, we anticipate that the use of fMRI to study rTMS in animal models will not only permit more detailed characterizations of how different rTMS protocols affect network dynamics and connectivity but will also elucidate how these changes reflect the manifestation of symptoms in preclinical models. Linking these functional changes to the molecular and cellular changes currently known in rodents will provide new insights into the fundamental mechanisms of brain plasticity and how to use rTMS therapeutically.

## Author contributions

BJS: Wrote and edited the manuscript; SE: Provided feedback, structured and edited the manuscript; KWF: Provided feedback, structured and edited the manuscript; JR: Conceived the idea and approach of the review, structured and edited the manuscript.

### Conflict of interest statement

The authors declare that the research was conducted in the absence of any commercial or financial relationships that could be construed as a potential conflict of interest.
